# Lack of vitamin D predicts impaired long-term immune response to COVID-19 vaccination

**DOI:** 10.1007/s12020-023-03481-w

**Published:** 2023-08-17

**Authors:** Luigi di Filippo, Stefano Frara, Umberto Terenzi, Fabrizio Nannipieri, Massimo Locatelli, Fabio Ciceri, Andrea Giustina

**Affiliations:** 1 Institute of Endocrine and Metabolic Sciences, San Raffaele Vita-Salute University and IRCCS Hospital, Milano, Italy; 2 Clinical Research, Abiogen Pharma, Pisa, Italy; 3 Laboratory Medicine Service, IRCCS San Raffaele Hospital, Milano, Italy; 4 Hematology and Bone Marrow Transplant Unit, San Raffaele Vita-Salute University and IRCCS Hospital, Milano, Italy

**Keywords:** Vitamin D, COVID-19, Anti-COVID-19 vaccine, Hypovitaminosis D, Anti-SARS-CoV-2 vaccine, SARS-CoV-2

## Abstract

**Purpose:**

Low vitamin D levels were reported to negatively influence the outcome of acute COVID-19, as well as to be linked to Long-COVID. However, few studies have investigated, so far, its effects on humoral-response to anti-SARS-CoV-2 vaccination, reporting conflicting results. We aimed to evaluate the impact of baseline 25(OH)vitamin D (25(OH)D) levels on humoral-response to a two-dose cycle of Pfizer-BioNTech-vaccine up to 9–10 months after immunization.

**Methods:**

We retrospectively included 119 consecutive healthcare-workers (median age 53 years) without a previous history of acute COVID-19 or anti-SARS-CoV-2 immunoglobulins presence immunized with two doses of Comirnaty-vaccine from January to February 2021. 25(OH)D was measured at time of first-immunization. Immune response was evaluated at: time 0 (T0), before the first-dose; T1, time of second-dose (21 days after T0); T2, T3, T4 at 1, 5 and 9 months after T1, respectively.

**Results:**

Median 25(OH)D levels were 25.6 ng/mL, and vitamin D deficiency (25(OH)D <20 ng/mL) was observed in 29 subjects (24.8%). In those with vitamin D deficiency, we found a non-significant trend towards lower antibody-titers at T3, and significantly lower titers at T4 as compared to those not vitamin D-deficient, also observing a more pronounced antibody-titers negative drop from peak-T2 and T4 in those with vitamin D deficiency. A positive correlation between 25(OH)D levels and antibody-titers at T4 (*p* = 0.043) was found. In multiple linear-regression analysis, 25(OH)D deficiency and older-age resulted as negative independent factors associated with antibody titer at T4 (*p* = 0.026, *p* = 0.004; respectively).

**Conclusion:**

In our relatively young cohort presenting low prevalence of hypovitaminosis D, the long-term humoral response to anti-SARS-CoV-2 vaccination was negatively influenced by low baseline 25(OH)D. Vitamin D supplementation could be tested as a strategy to optimize the vaccination campaigns to prevent severe COVID-19.

## Introduction

Vitamin D is known to regulate the immune system activity [1–4] including possible impact on humoral response to vaccinations which was studied in animal models and humans mostly reporting positive influences [1, 5]. Few studies have investigated so far the effects of vitamin D status and chronic supplementation on humoral response after anti-SARS-CoV-2 vaccination, reporting conflicting results probably due to heterogeneous study groups and length of follow-up [6–8]. The aim of this study was to evaluate the impact of baseline 25(OH) vitamin D (25(OH)D) levels on humoral response to a two-dose cycle of Comirnaty (BNT162b2, Pfizer-BioNTech) vaccine in a cohort of healthcare workers with no previous history of COVID-19 followed-up for 9–10 months after immunization.

## Methods

### Study design

We retrospectively evaluated data of consecutive adult healthcare subjects immunized with two doses of Comirnaty vaccine from January to February 2021. This work is a cohort sub-study part of an institutional monocentric prospective observational research, the COVID-BioVac study, carried out at IRCCS Ospedale San Raffaele, a tertiary health care center in Milan, Italy (Latitude: 45.464664), and was specifically approved by the local hospital ethics committee (protocol ABIO/NC/05 no. 157/2022). Signed informed consent was obtained from all individuals participating in this study.

**Fig. 1:**
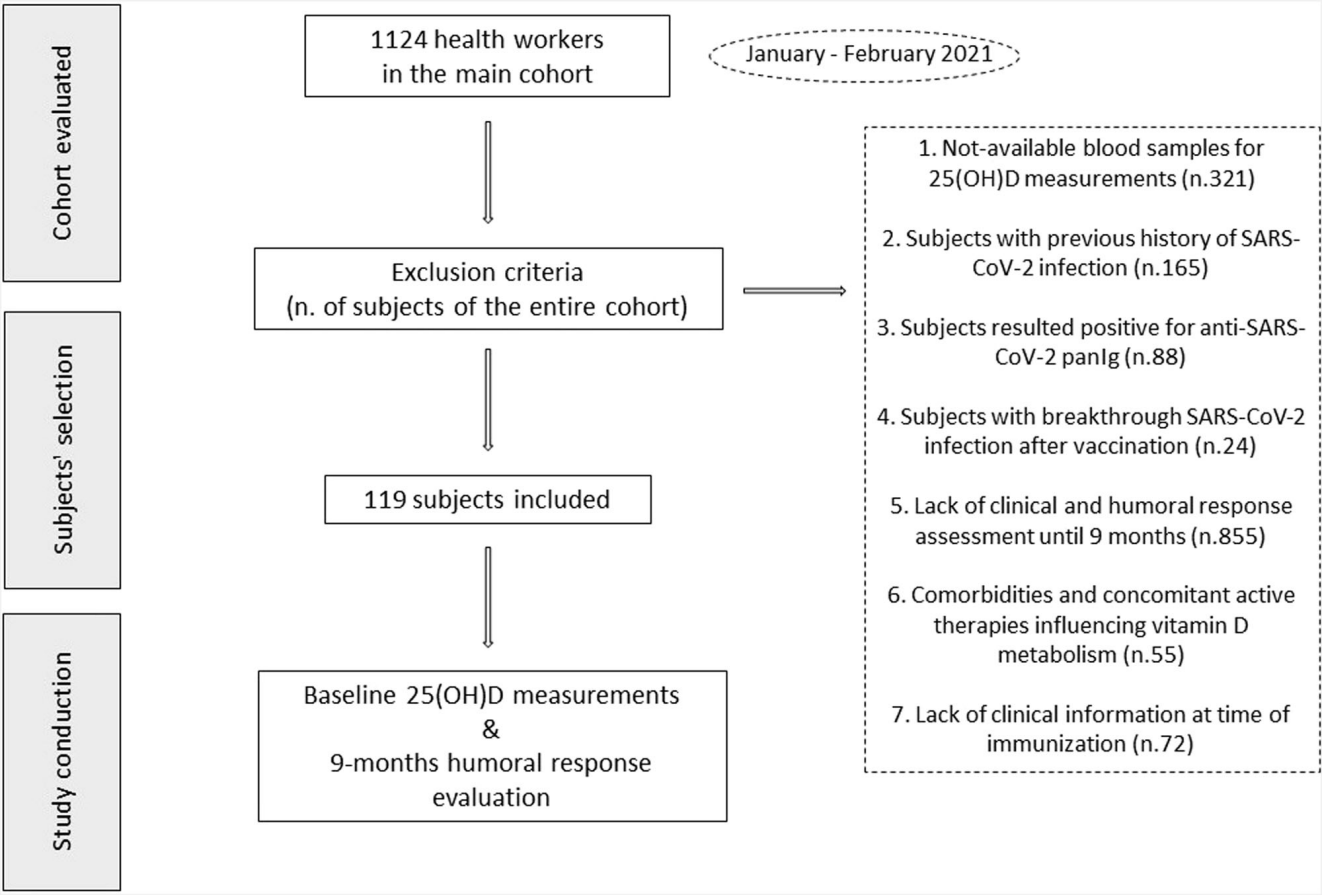
Study design and retrospective enrollment flow chart

Among the health workers participating to the institutional study, we retrospectively included in this sub-study only subjects without either a previous history of SARS-CoV-2 infection or COVID-19 symptoms, and those without immunoglobulins anti-SARS-CoV-2 at time of first immunization dose. Only individuals with available medical data recorded upon date of first vaccine dose administration were included in this study. Subjects with the following comorbidities and concomitant active therapies influencing vitamin D metabolism were excluded: chronic kidney disease, active neoplasia, osteoporosis, subjects on chronic glucocorticoids and antiepileptic drugs, vitamin D/calcium supplements, and loop/thiazide diuretics. Also, we did not retrospectively include in the study subjects for whom breakthrough SARS-CoV-2 infections after the vaccination was reported in the database. Based on these inclusion and exclusion criteria 119 subjects were finally included in our study. The study design and the enrollment flow chart are summarized in Fig. 1.

### Data and antibodies collection

25(OH)D was measured on blood samples collected at time of first immunization in retrospectively enrolled subjects with Roche 10 Cobas 8000 WKC/MET/036 electrochemiluminescence immunoassays (coefficient of variation of 5%) (ng/mL). Vitamin D deficiency was defined as 25(OH) vitamin D levels below 20 ng/mL, according to the cut-off values reported by Sempos et al. [9]. Body mass index (BMI) (calculated as the ratio of weight in kilograms divided by height in meters squared) was collected for all subjects. Overweight was defined as a BMI ≥ 25 kg/m^2^, and obesity as BMI ≥ 30 kg/m^2^. Data from obese and overweight subjects were analyzed together.

Immune response was evaluated at: time 0 (T0), before the first vaccination; T1, time of second vaccination (21 days after T0); T2, T3, T4 at 1, 5 and 9 months after T1, respectively.

At T0, the blood samples were tested for the presence of pan-Immunoglobulins (pan-Ig: IgA, IgG and IgM) against the SARS-CoV-2 viral nucleocapsid protein (N-protein) to detect subjects previously infected. This test was performed using the Roche Anti-SARS-CoV-2, ECLIA (Sensitivity: 100%; Specificity: 99.8%, by adopting the manufacturer’s suggested cutoff of 1 U/mL), on a COBAS 601 platform (Roche, Basel, Switzerland) [10–12].

At T1, T2, T3 and T4, the blood samples were tested for the presence of antibodies against the receptor binding domain (RBD) of the viral S-protein (anti-RBD-S IgTot) to assess the immune responses to vaccination. These tests were performed using the Roche ECLIA anti-SARS-CoV-2-S test (Roche, Basel, Switzerland). The quantification range was between 0.4 and 250.0 U/mL, which was further extended to 2500.0 U/mL by a 1:10 dilution of the sample automatically performed by the instrument. Specificity and sensitivity are 99.98% and 98.8%, respectively, by adopting the manufacturer’s suggested cutoff of 0.8 U/mL. Roche declared that the conversion factor between “anti-SARS-CoV-2-S” test results expressed in U/mL and Binding antibody units per milliliter (BAU/mL) proposed by the WHO is 1. Thus, test results expressed in U/mL throughout the paper correspond to BAU/mL [10–12].

## Results

One-hundred nineteen subjects were retrospectively enrolled. Median age (interquartile range) was 53 (33–57) years and 61 were males (51.3%). The most frequent concomitant comorbidity in this cohort was history of hypertension (16%). Median BMI was 23.4 (21.5–25.9), and 15 individuals (12.6%) were obese and 37 (31%) were overweight/obese. Twelve (10.1%) subjects were past-smokers and 20 (16.8%) were active-smokers.

Median 25(OH)D levels were 25.6 ng/mL (20–32), and vitamin deficiency was observed in 29 subjects (24.8%). Between those with hypovitaminosis D, a severe vitamin D deficiency (25(OH)D < 12 ng/mL) was found in only two out of 29 subjects (6.9%). No statistically significant differences were observed regarding age, BMI, history of past-smoking and active-smoking between subjects with vitamin D deficiency and those without. No subject was taking vitamin D supplements either at baseline or during the study.

At T0, as per protocol, no one was seropositive for anti-SARS-CoV-2 N-protein pan-Ig. At T1, 21 days after T0, we observed a median anti-RBD-S IgTot titer of 42.5 U/mL (17.4–109). At T2, 1 month after T1, we observed a median anti-RBD-S IgTot titer of 2024 U/mL (1186–2500). At T3, 5 months after T1, we observed a median anti-RBD-S IgTot titer dropped to 710 U/mL (456–1131). At T4, 9 months after T1, the median anti-RBD-S IgTot titer value decreased to 507 U/mL (284–769), consistent with a 75–80% drop compared to the estimated peak at T2.

**Fig. 2:**
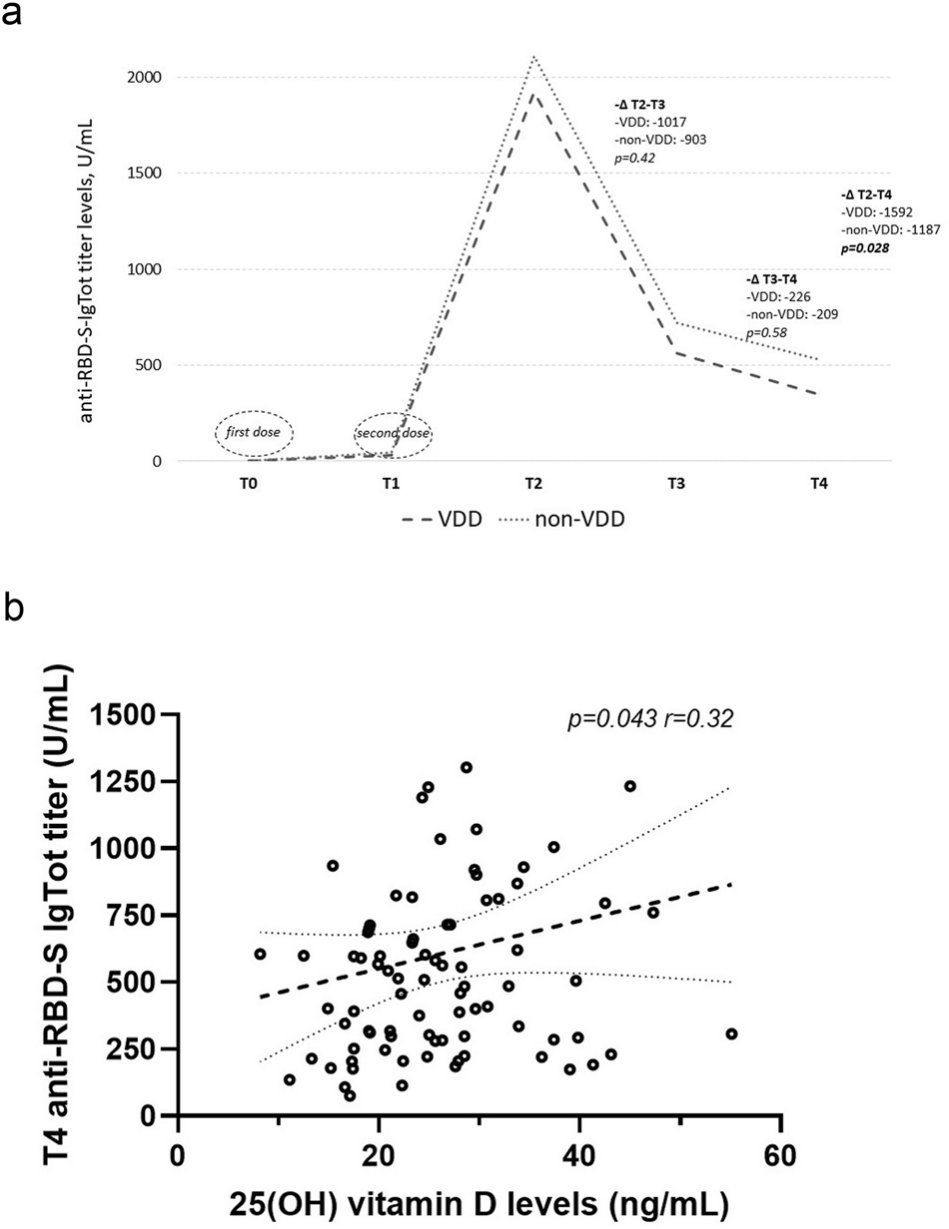
**a** Anti-RBD-S-IgTot titers negative drop (-∆) from T2 peak and T3, and T4, in subjects with vitamin D deficiency (VDD) and those without (non-VDD). **b** Linear correlation between 25(OH) vitamin D levels and anti-RBD-S IgTot titer at 9–10 months after vaccination (T4)

In subjects with vitamin D deficiency, we found a non-significant trend towards lower anti-RBD-S-IgTot titers at T3, and significantly lower anti-RBD-S-IgTot titers at T4 as compared to those not vitamin D-deficient (Table 1), also observing a more pronounced anti-RBD-S-IgTot titers negative drop from peak-T2 and T4 in vitamin D deficient subjects (Fig. 2a). A positive correlation between 25(OH)D levels and anti-RBD-S-IgTot titers at T4 (*p* = 0.043, *r* = 0.32) was found (Fig. 2b).

**Table 1:** Comparisons regarding anti-RBD-S-IgTot titer levels and respective negative drops between subjects with and without vitamin D deficiency at the different timepoints

	Vitamin D deficient (n.29)	Non-Vitamin D deficient (n.90)	*P* value
T1 IgTot, U/mL	30.85 [15.9–119.2]	44.2 [18.5–109]	0.56
T2 IgTot, U/mL	1925 [1167–2500]	2108 [1180–2500]	0.52
T3 IgTot, U/mL	564 [389.5–980]	720 [467–1134]	0.081
T4 IgTot, U/mL	346 [191.5–598]	528 [298–822.5]	**0.023**
∆ T2-T3, U/mL	−1017 [524–1437]	−903 [361–1520]	0.42
∆ T3-T4, U/mL	−226 [137–429]	−209 [96–422]	0.58
∆ T2-T4, U/mL	−1592 [1217–1874]	−1187 [587–1781]	**0.028**

Bold values identify statistical significance.

No statistically significant differences were observed regarding T1-T2-T3-T4 IgTot titers among overweight/obese vs non-overweight/obese and obese vs non-obese subjects; and among past-smokers vs non-past-smokers and active-smokers vs non-active smokers. No significant correlations were observed between BMI and IgTot titers at any timepoints.

In multiple linear regression analysis, 25(OH)D deficiency and age resulted as negative independent factors associated with anti-RBD-S-IgTot titer at T4 (*p* = 0.026, standardized beta −0.35; *p* = 0.004, standardized beta −0.44; respectively).

## Discussion

During the pandemic spread, increasing evidence has highlighted the potential role of hypovitaminosis D as a modifiable risk factor for SARS-CoV-2 infection and worse acute COVID-19 [3, 13–16]. Furthermore, lower vitamin D levels were also recently associated to an increased risk for the occurrence of Long COVID syndrome [17].

Due to the well-known actions of vitamin D in regulating the immune response and immunocompetence [18, 19] previous studies have investigated its role also in influencing the humoral response after different vaccinations [1, 5]. In our study, the long-term humoral response to anti-SARS-CoV-2 vaccination clearly appeared to be influenced by baseline 25(OH)D levels. In fact, we found that poor vitamin D status, as defined by 25(OH)D levels <20 ng/mL [20], before the immunization was associated with reduced anti-RBD-S-IgTot levels 5 and 9 months after vaccination. Differently from most of the previously published studies, our findings were controlled in nature since in our work stringent inclusion and exclusion criteria were used enrolling a homogenous population of healthcare workers immunized in the same time period with the same vaccine type, and including only individuals who were not supplemented with vitamin D and naïve for SARS-CoV-2 infection and without breakthrough infections during the study-period. Furthermore, subjects were followed-up for a much longer time period as compared to the previously available studies. This allowed us to focus not only on the impact of vitamin D levels on peak humoral response but also on the protracted and long-term efficacy of anti-SARS-CoV-2 vaccination. Clinically, this may be a relevant information since subjects with reduced antibody persistence should likely require more frequent additional booster vaccinations to preventing COVID-19 worse outcomes. Interestingly, we observed that an adequate vitamin D status at the beginning of the immunization cycle was associated with a significantly more sustained anti-SARS-CoV-2 antibody response.

The retrospective nature of our study did not allow us to assess the protective effects of the persistence of higher antibody titers against either SARS-CoV-2 infection or severe acute COVID-19. In fact, on one hand, anti-SARS-CoV-2 antibody titers are tightly linked to degree of protection at least against severe forms of COVID-19 [21, 22]. On the other hand, in fragile and high-risk populations, such as elderly, patients with cancers or under dialytic treatment, characterized by an immune response impairment with reduced humoral response, despite poor seroconversion in comparison to healthy subjects, the protective effect of the vaccine seems to be maintained [23–25]. Nevertheless, in support of clinical relevance of our data, it is also widely recommended in these fragile populations to administer extra- and booster vaccine doses to grant them an adequate protection against the severe infection [26–29] suggesting that it is not the short-term but the persistency of the response to the vaccine to be impaired.

It could be also hypothesized that hypovitaminosis D may represent a marker of limited outdoor activities and fragility, possibly in turn being per se, at least in part, responsible for the lower observed antibody levels [30]. However, no one of our study subjects could be defined “fragile” or with intrinsic high-risk for impaired immune response, since we included in our cohort only healthy and relatively young health hospital workers. Also, the lack of 25(OH)D levels re-evaluation at different study-timepoints could represent a limitation of the study, although the possible effect of seasonality on 25(OH)D levels should apply to all subjects included in the study due to the very short time-window of enrollment. Moreover, other data, beyond BMI, about the metabolic features of our subjects were not available since not included in main study protocol. Finally, our data were obtained in a very selected relatively young cohort without a high prevalence of hypovitaminosis D and therefore could not be automatically extended to the general population in which however the reported scenario may even be worsened in case of more generalized and severe vitamin D deficiency.

Besides these limitations, our data showed that people with vitamin D deficiency were at risk of a less sustained immune response to COVID-19 vaccine. Vitamin D deficiency was also extensively reported as an important modifiable risk factor for severe COVID-19 increasing the risk of worse outcomes [3, 13–16]. Severe COVID-19 was reportedly associated with post-infection elevated peak of antibodies against SARS-CoV-2 [31, 32] which has been hypothesized, although not yet proven, to occur regardless of vitamin D status [33]. Interestingly, a limited influence of hypovitaminosis D on this short-term humoral response is also observed in our data and in the previous published reports, supporting the hypothesis that vitamin D levels may be associated with the long-term humoral response to COVID-19 vaccine rather than to the acute seroconversion, elicited by the infection and/or vaccination.

In conclusion, in a cohort of highly selected relatively young subjects with limited prevalence of hypovitaminosis D, low levels of circulating 25(OH)D were associated with impaired long-term response to COVID-19 vaccine. Therefore, based on our data it can be hypothesized that at least in populations at higher risk of both hypovitaminosis D and COVID-19 such as the elderly [34], assessing vitamin D levels and eventually improving a poor vitamin D status before the vaccination could be a possible strategy to optimize the vaccination campaigns to prevent severe COVID-19. This hypothesis needs to be tested in controlled clinical trials assessing the effect of vitamin D supplementation in subjects with hypovitaminosis D on the long-term vaccine protection against worse COVID-19 outcomes and the requirement for vaccine boosts which may have both clinical and economic favorable implications.

## Data Availability

All authors had full access to all the data in the study and takes responsibility for the integrity of the data and the accuracy of the data analysis.
